# *Mycoplasma pneumoniae*‐Induced Rash and Mucositis: Clinicopathologic Characterization of 11 Cases

**DOI:** 10.1111/cup.70028

**Published:** 2025-11-26

**Authors:** Margaret Lang Houser, Jennifer B. Mancuso, Johann E. Gudjonsson, Alexandra C. Hristov, Lori Lowe, May P. Chan

**Affiliations:** ^1^ Department of Dermatology University of Michigan Ann Arbor Michigan USA; ^2^ Department of Pathology University of Michigan Ann Arbor Michigan USA

**Keywords:** erythema multiforme (EM), *Mycoplasma pneumoniae* ‐induced rash and mucositis (MIRM), reactive infectious mucocutaneous eruption (RIME), Stevens–Johnson syndrome

## Abstract

**Background:**

*Mycoplasma pneumoniae* ‐induced rash and mucositis (MIRM) is a mucocutaneous eruption affecting children and young adults with respiratory tract or clinically occult infection by *M. pneumoniae* . Mucosal involvement is often robust and may raise concern for Stevens–Johnson syndrome/toxic epidermal necrolysis (SJS/TEN). Histopathologic changes in MIRM have not been systematically evaluated.

**Methods:**

Eleven cases of clinically and serologically confirmed MIRM with biopsies available were included in this study. Clinical and histopathologic features were reviewed and recorded.

**Results:**

All cases displayed a vacuolar to lichenoid interface reaction with apoptotic keratinocytes or cytoid bodies confined to the epidermis. Subepithelial split and epithelial necrosis were observed in about half of the cases, some of which closely mimicked SJS/TEN histopathologically. There was a predominance of neutrophils over lymphocytes in the lichenoid infiltrate in a small subset of cases, a finding that was associated with leukocytosis and concomitant disease involvement of skin and all three mucosal sites.

**Conclusions:**

The majority of MIRM cases demonstrated histopathologic features indistinguishable from those of erythema multiforme or SJS/TEN, with the exception of a neutrophil‐rich lichenoid infiltrate observed in a small subset of cases. MIRM is essentially synonymous with erythema multiforme major associated with *M. pneumoniae* infection. Correlation with clinical and serologic findings is necessary to exclude SJS/TEN.

## 1 Introduction

*Mycoplasma pneumoniae* is a common cause of mild respiratory tract infection. Mucocutaneous eruption associated with *M. pneumoniae* infection is a relatively uncommon manifestation, reported in 11%–13% of patients hospitalized for *M. pneumoniae* [1]. This phenomenon preferentially affects the pediatric population and was termed *Mycoplasma pneumoniae* ‐induced rash and mucositis (MIRM) by Canavan et al. in 2015 [2]. Clinically it is characterized by prominent mucosal involvement and usually sparse cutaneous lesions. Most patients report prodromal symptoms including cough, fever, and malaise. Oral mucosa, conjunctiva, and the urogenital tract are frequently affected and may raise clinical concern for Stevens–Johnson syndrome/toxic epidermal necrolysis (SJS/TEN). Cutaneous lesions commonly present as vesicles or targetoid papules. The prognosis of MIRM is generally excellent with most patients experiencing full recovery without complications.

Although MIRM differs from erythema multiforme by the severity of mucosal involvement (usually involving two or more mucosal sites) and paucity of cutaneous lesions, the distinction between the two entities remains somewhat poorly defined and controversial. Reactive infectious mucocutaneous eruption (RIME) is a more encompassing term used to describe parainfectious mucocutaneous reactions triggered by a variety of bacterial and viral diseases, including SARS‐CoV‐2, influenza A and B, adenovirus, *Chlamydia pneumoniae* , and *M. pneumoniae* , among many others. MIRM therefore falls under the rubric of RIME [3, 4, 5, 6, 7, 8, 9, 10, 11].

Additional case reports and case series have deepened our understanding of the clinical presentations of MIRM; however the histopathologic changes remain underreported. Apoptotic keratinocytes, epidermal necrosis, and lichenoid infiltrate were the most commonly reported features among cases with documented biopsy findings [12, 13, 14]. Herein we conducted a comprehensive histopathologic review of clinically and serologically confirmed MIRM cases, in order to facilitate accurate clinicopathologic diagnosis of this unique disease.

## 2 Materials and Methods

After approval by the Institutional Review Board, the electronic medical records at the University of Michigan were searched for “ *Mycoplasma pneumoniae* ‐induced rash and mucositis,” “MIRM,” “reactive infectious mucocutaneous eruption,” and “RIME” from 2013 to 2024 using the Electronic Medical Record Search Engine (EMERSE), an information retrieval system [15]. Cases with biopsy results and a final leading diagnosis of MIRM were included in the study. Detailed clinical history was obtained from specimen requisitions and/or clinic notes. Relevant laboratory data, including serologies of IgG and IgM antibodies against *M. pneumoniae* , and evidence of leukocytosis (white blood cell count > 10.0 K/μL) or neutrophilia (absolute neutrophil count > 7.2 K/μL), were gathered from electronic medical records. Each skin biopsy was assessed for a variety of histopathologic features by a board‐certified dermatopathologist. Potential associations between different parameters were evaluated by student *t* tests (continuous data) or Chi square tests (categorical data). A *p* value of less than 0.05 was considered statistically significant.

## 3 Results

**FIGURE 1:**
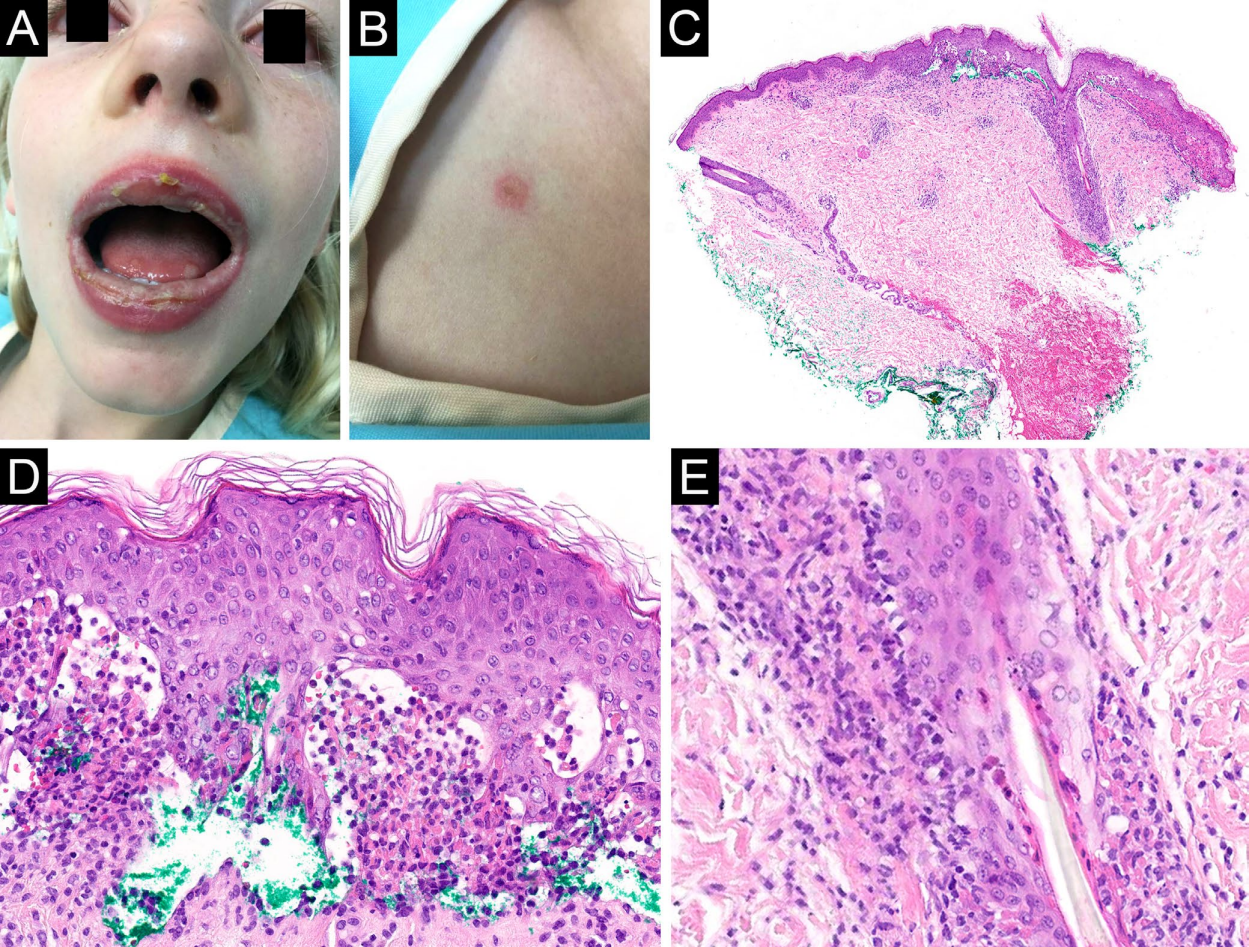
*Mycoplasma pneumoniae* ‐induced rash and mucositis, Case 1. (A) Bilateral conjunctival injection, desquamation of the lips, and erosions on the distal tongue. (B) An erythematous papule with central duskiness on the chest. (C) Skin biopsy reveals a lichenoid interface dermatitis with a subepidermal split (H&E, 20×). (D) Numerous neutrophils and cytoid bodies are found within the subepidermal cleft (H&E, 400×). (E) The lichenoid infiltrate and interface changes are found in the follicular epithelium as well (H&E, 400×).

**FIGURE 2:**
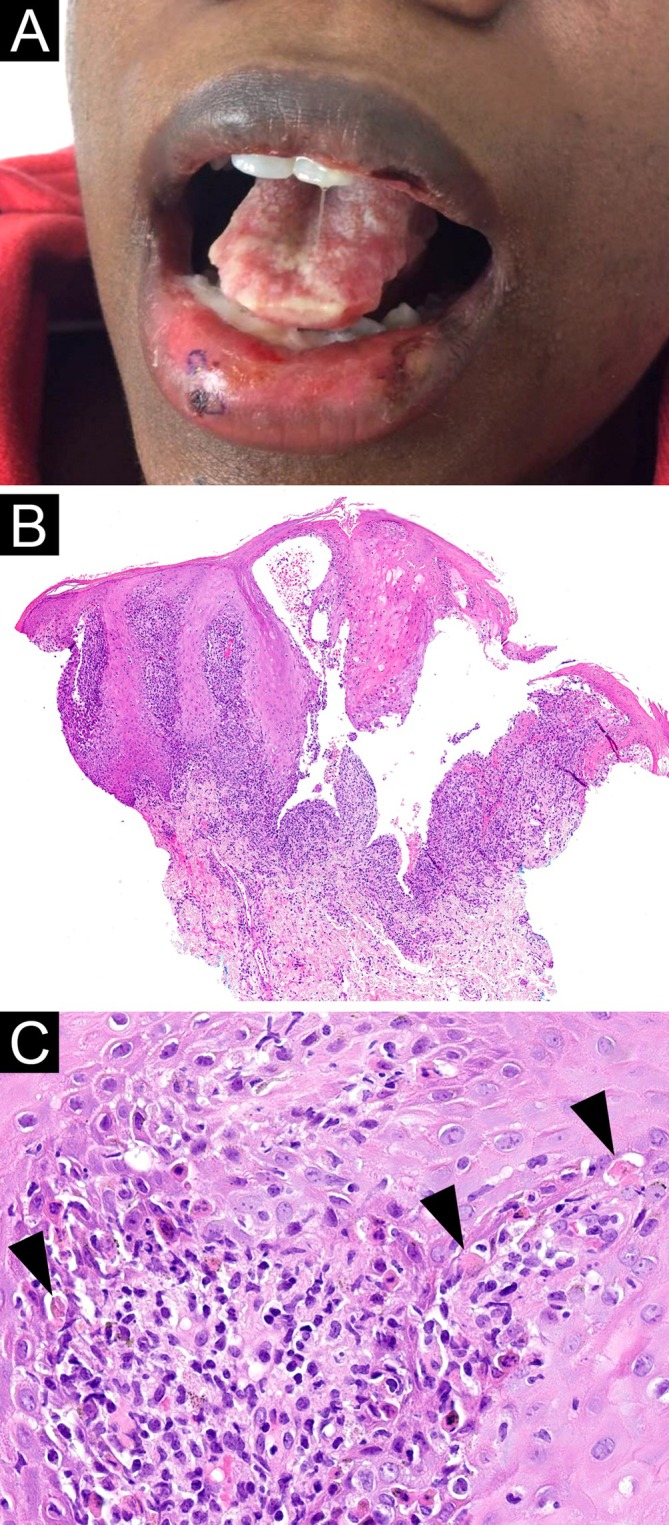
*Mycoplasma pneumoniae* ‐induced rash and mucositis, Case 2. (A) Erosions on the mucosal and cutaneous lips, and friable white plaques on the dorsal and lateral tongue with areas of erosion. (B) A lip biopsy shows a dense lichenoid infiltrate involving the hyperplastic mucosal epithelium with subepithelial clefting (H&E, 20×). (C) The lichenoid infiltrate consists of lymphocytes and scattered neutrophils. Many apoptotic keratinocytes are present in the basal epithelium (arrowheads) (H&E, 400×).

**FIGURE 3:**
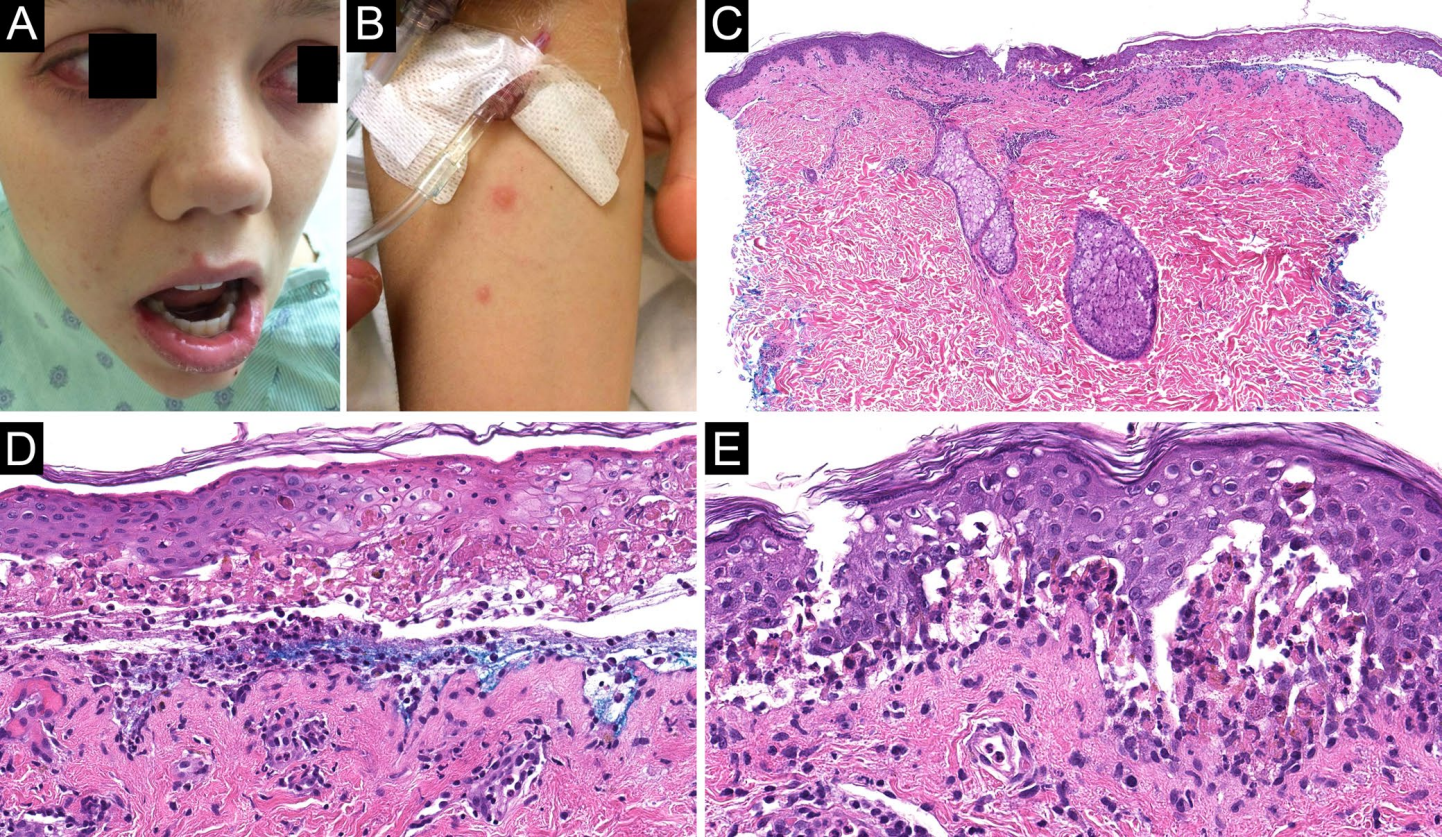
*Mycoplasma pneumoniae* ‐induced rash and mucositis, Case 3. (A) Conjunctival injection and a superficial erosion on the right lower mucosal lip. This patient also had many superficial erosions on the palate and posterior pharynx. (B) Sparse erythematous papules on the right arm (H&E, 20×). (C) A subepidermal split with partial necrosis of the detached epidermis. (D) Numerous cytoid bodies confined to the undersurface of the blister roof (H&E, 200×). (E) The inflammatory infiltrate at the dermoepidermal junction is composed predominantly of neutrophils (H&E, 400×).

**FIGURE 4:**
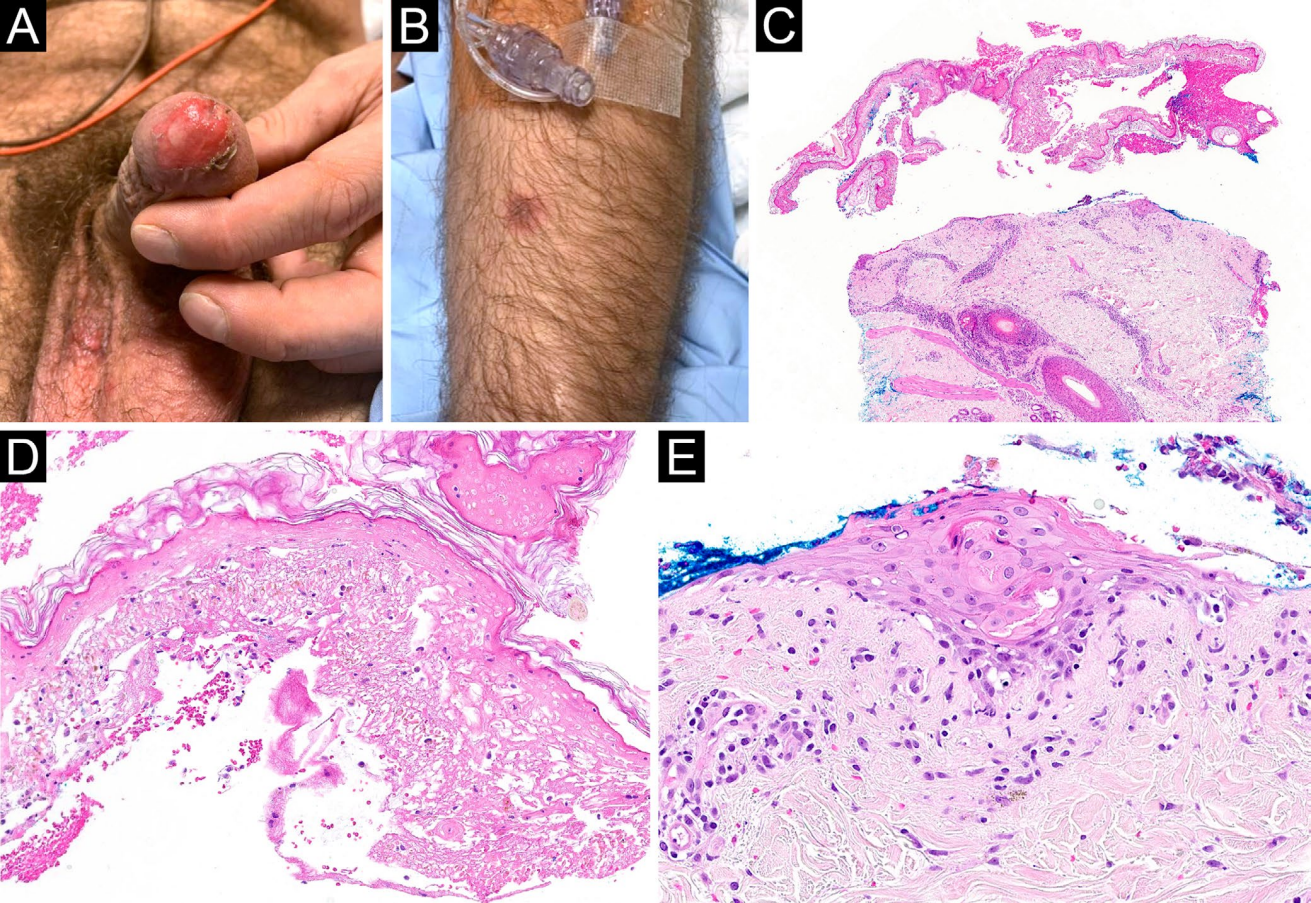
*Mycoplasma pneumoniae* ‐induced rash and mucositis, Case 5. (A) A ruptured blister on the glans penis with involvement of the urethral meatus. (B) A purpuric macule on the left arm. (C) Biopsy of the left arm lesion shows a pauci‐inflammatory subepidermal blister closely resembling Stevens–Johnson syndrome (H&E, 20×). (D) The blister roof is completely necrotic (H&E, 200×). (E) Close inspection of the blister floor reveals focal intact epidermis with mild vacuolar interface dermatitis (H&E, 200×).

**FIGURE 5:**
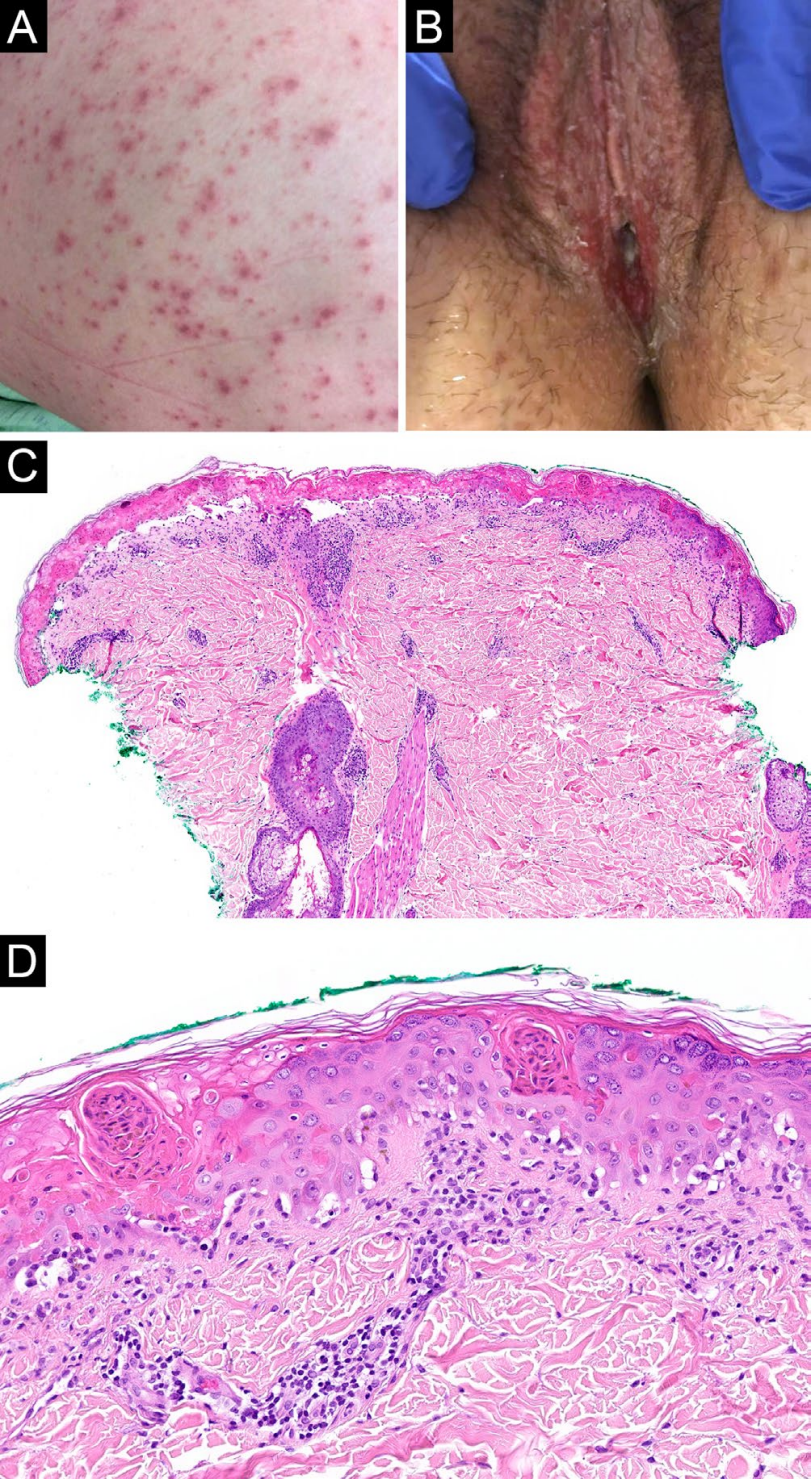
*Mycoplasma pneumoniae* ‐induced rash and mucositis, Case 10. (A) Numerous erythematous macules, some with a dusky center. (B) Erosions and swelling of bilateral labia. (C) A skin biopsy reveals partial epidermal necrosis and a perifollicular inflammatory infiltrate (H&E, 20×). (D) Higher magnification shows a vacuolar interface dermatitis with many cytoid bodies confined to the epidermis (H&E, 100×).

Eleven cases meeting inclusion criteria were identified. Clinical and laboratory findings are summarized in Table 1. All patients were children and young adults aged 7–35 years (median, 21 years) with a female‐to‐male ratio of 6:5. Oral lesions were present in all cases, typically described as crusted erosions, and most frequently involving the lips and less frequently the buccal mucosae, palate, tongue, and gingiva (Figures 1, 2, 3). Conjunctivitis and urogenital lesions were reported in 6 (55%) and 5 (45%) patients, respectively (Figures 1 and 3, 4, 5). Cutaneous lesions were seen in 8 (73%) patients, presenting as targetoid papules or plaques on the extremities (including palms and soles), trunk, or face (Figures 1 and 3, 4, 5), with an average involved body surface area of 5% (range, 1%–9%). Preceding or concurrent pneumonia, upper respiratory infection, and/or systemic symptoms were documented in 5 (45%) patients. All patients tested positive/reactive for IgM antibodies to *M. pneumoniae* . Blood counts showed leukocytosis and neutrophilia in 2 (18%) and 5 (45%) patients, respectively. None of the patients developed systemic or cutaneous lupus erythematosus before or after the diagnosis of MIRM. Antinuclear antibody was negative in all five patients tested.

**TABLE 1:** Clinical and laboratory data.

Case	Age/sex	Systemic/respiratory symptoms	Sites of MIRM				Laboratory workup		
			Oral	Conjunc‐tiva	Uro‐genital	Skin (BSA)	*M. pneumoniae* IgM	Leukocytosis	Neutrophilia
1	7/F	+	+	+	+	+ (1%)	+	+	+
2	23/F	−	+	−	−	−	+	−	−
3	21/F	+	+	+	+	+ (1%)	+	+	+
4	17/F	−	+	+	−	−	+	n/a	n/a
5	35/M	+	+	+	−	+ (5%)	+	−	+
6	9/M	+	+	+	+	+ (5%)	+	−	−
7	25/F	−	+	−	−	+ (2%)	+	n/a	n/a
8	22/M	−	+	+	+	+ (5%)	+	−	+
9	15/M	+	+	−	−	−	+	−	−
10	19/F	−	+	−	+	+ (9%)	+	−	+
11	33/M	−	+	−	−	+ (9%)	+	−	−

*Note*: −, absent; +, present.

Abbreviations: BSA, body surface area involved; F, female; M, male; MIRM, *Mycoplasma pneumoniae* ‐induced rash and mucositis; n/a, not available.

Microscopic features of the 11 biopsies are summarized in Table 2. Four biopsies were taken from mucosal lips; the remaining biopsies were taken from various skin sites. All biopsies featured a vacuolar or lichenoid interface dermatitis (Figures 1, 2, 3, 4, 5), although such findings were focal and subtle in Case 4. Subepithelial split was seen in 6 (55%) biopsies, with epithelial necrosis in 5 (45%) cases (Figures 1, 2, 3, 4, 5). Cytoid bodies and apoptotic keratinocytes were numerous in 7 (64%) cases, found exclusively within the epithelium or in the subepithelial blister cavity; none were identified in the underlying dermis or lamina propria (Figures 1, 3, and 5). The interface changes and the inflammatory infiltrate extended along follicular or eccrine epithelia in 6 (75%) of the 8 skin biopsies containing adnexal structures (Figures 1, 4, and 5).

**TABLE 2:** Biopsy information and histopathologic findings.

	Cases										
	1	2	3	4	5	6	7	8	9	10	11
Site of biopsy	Medial thigh	Lower lip	Back	Lower lip	Arm	Arm	Palm	Digit	Lower lip	Back	Digit
Days since onset	3	3	4	3	2	7	NA	7	10	3	13
Primary pattern	V/L	L	V	V	V	V	L	V	L + Sp	V/L	V/L
Apoptotic/cytoid bodies	3+	2+	3+	0	3+	3+	3+	3+	1+	3+	2+
Basal vacuolization	0	1	1	1	1	1	1	1	0	1	1
Lymphocytes at interface	2+	3+	1+	1+	2+	2+	2+	1+	2+	2+	3+
Neutrophils at interface	3+	1+	3+	0	0	1+	0	0	0	0	0
Subepithelial split	1	1	1	0	1	0	0	1	0	1	0
Epithelial necrosis	1	0	1	0	1	0	0	1	0	1	0
Parakeratosis	0	1	0	0	0	0	1	1	0	1	1
Adnexal inflammation	1+	0	1+	0	1+	2+	0	0	0	1+	2+
Perivascular lymphocytes	S	S	S	S	S	S	S	S	S + D	S	S
Perivascular eosinophils	0	0	0	0	0	0	0	0	0	0	0
Direct immunofluorescence	ND	−	−	−	−	ND	ND	ND	−	ND	ND

*Note*: −, negative; 0, absent; 1, present; 1+, sparse; 2+, moderate; 3+, brisk.

Abbreviations: D, deep; L, lichenoid; NA, not available; ND, not done; S, superficial; Sp, spongiotic; V, vacuolar.

Two (18%) biopsies demonstrated a predominance of neutrophils over lymphocytes in the lichenoid infiltrate (Figures 1 and 3). Another 2 (18%) biopsies contained a minor population of neutrophils (Figure 2). The neutrophils spared the upper epithelium and the stratum corneum, thus speaking against a bacterial superinfection. The remaining (64%) cases were lymphocytic and largely devoid of neutrophils (Figures 4 and 5). The presence of neutrophils was associated with fever at the time of presentation (*p* = 0.04). Furthermore, the predominance of neutrophils was significantly associated with leukocytosis (*p* = 0.003) but not with neutrophilia (*p* = 0.15). Patients with neutrophil‐rich biopsies were more likely to have concomitant involvement of skin and all three mucosal (oral, conjunctival, and urogenital) sites (*p* = 0.04), although there was no correlation with the extent of skin involvement. The presence or predominance of neutrophils did not correlate with the timing of biopsy (*p* = 0.27), and did not give rise to a distinct clinical morphology of the lesions.

Dermal inflammation was typically mild and perivascular, consisting of lymphocytes without admixed eosinophils. Deep dermal perivascular lymphocytes were only seen in 1 (9%) case. Intravascular neutrophilic margination was noted in 2 (18%) cases.

Five patients underwent a second biopsy for direct immunofluorescence studies, all of which were negative for specific immune deposition.

## 4 Discussion

The histopathology of MIRM has been understudied. Previously reported cases focused on the clinical presentation of the mucocutaneous eruption. Biopsy findings were either not discussed or briefly described as “epidermal necrosis,” “apoptotic keratinocytes,” and “lichenoid infiltrate” [12, 13, 14]. While a common assumption was an erythema multiforme‐like histomorphology in MIRM, a systematic analysis was lacking. Here we report the histopathologic findings in 11 skin and mucosal biopsies from patients diagnosed with MIRM based on clinical presentations and positive *Mycoplasma* serology.

Our series confirmed that MIRM is a vacuolar to lichenoid interface process. The intensity of inflammation involving the surface epithelium and the degree of basal vacuolization ranged from mild to robust. Apoptotic keratinocytes or cytoid bodies were common findings, except in Case 4 where the inflammatory infiltrate was sparse and caused only basal vacuolization. Interestingly, cytoid bodies were confined to the epithelium or subepithelial cleft, and were not found in the underlying dermis or lamina propria, similar to erythema multiforme and SJS/TEN. Subepithelial split and epithelial necrosis were observed in about half of the cases, although these findings are presumably highly dependent on the type of lesions being biopsied. While MIRM is a mucosal‐predominant disease, there is likely a tendency to avoid biopsy of the mucosal erosions when cutaneous lesions are present. Furthermore, sampling of clinically eroded, bullous, or dusky lesions is expected to show subepithelial split and/or epithelial necrosis, compared to those with only erythema. Importantly, cases with extensive epidermal necrosis and sparse inflammation (such as Case 5) can closely mimic SJS/TEN microscopically. Correlation with clinical findings, including the paucity of cutaneous lesions and the absence of a preceding new medication, is key in excluding SJS/TEN in such cases.

While lymphocytes were the predominant cell type in most cases, an interesting finding is the presence of neutrophils in the lichenoid infiltrate in a minority of cases. This was particularly prominent in Cases 1 and 3, in which neutrophils predominated over lymphocytes. To our knowledge, this feature has only been reported in one RIME case secondary to COVID‐19^7^ but has not been previously associated with *M. pneumoniae* . In our series, the presence of neutrophils did not correlate with any distinctive lesional morphology, blood neutrophilia, extent of skin involvement, or time lapse between onset of mucositis and biopsy. This contrasts with so‐called neutrophilic fixed drug eruption, in which a prominent neutrophilic infiltrate is believed to represent the earliest phase of this lichenoid interface dermatitis [16, 17]. Interestingly, both of our patients with leukocytosis displayed brisk neutrophils on biopsy, and concomitant involvement of skin and all three mucosal sites. Patients with neutrophils on biopsy were also more likely to be febrile. Collectively, these associations point to neutrophils as a potential marker of a more robust reaction to *Mycoplasma* infection. While the combination of subepithelial split and neutrophilic inflammation may raise consideration for an autoimmune bullous disorder such as linear IgA bullous dermatosis, dermatitis herpetiformis, or bullous lupus erythematosus, the abrupt onset of the rash and the striking preferential involvement of mucosal surfaces, as well as microscopic evidence of interface dermatitis would speak against these. Direct immunofluorescence may be necessary when this dilemma cannot be readily resolved.

Adnexal involvement by vacuolar interface dermatitis was a relatively common finding in our cohort, raising brief consideration for cutaneous lupus erythematosus, especially in light of the association between *Mycoplasma pneumoniae* infection and the development of systemic lupus erythematosus [18]. However, none of these patients went on to develop lupus or tested positive for antinuclear antibody, speaking against a cutaneous manifestation of lupus erythematosus.

Our histopathologic observations largely corroborate the common belief that MIRM and erythema multiforme exist on the same disease spectrum. In particular, MIRM shares many clinical features with erythema multiforme major, a variant with significant mucosal involvement. Of the microscopic findings identified in our series, only neutrophilic lichenoid infiltrate is unique to MIRM and has not been described in erythema multiforme. Interestingly, a previous study has found that erythema multiforme triggered by *M. pneumoniae* was associated with more severe mucositis and atypical targetoid skin lesions compared to erythema multiforme associated with other pathogens (most commonly herpes simplex virus). This study also found that histopathologically, *M. pneumoniae* ‐related erythema multiforme frequently mimicked SJS/TEN with epidermal necrosis. Integrating all findings, *M. pneumoniae* ‐associated erythema multiforme and MIRM are likely the same disease, with only semantic differences.

Our cohort is limited by the small number of cases owing to the rarity of MIRM cases with serologic confirmation and biopsies. The paucity of mucosal biopsies in this retrospective study, as discussed above, also limits the representation of mucosal lesions. Finally, although there are no previous reports of neutrophils in erythema multiforme or SJS/TEN in the literature, we did not directly examine these entities for comparison. The link between neutrophils and MIRM or RIME therefore remains to be further elucidated.

In summary, our series provides a comprehensive characterization of the histopathologic changes in MIRM, consistently featuring a vacuolar or lichenoid interface reaction involving variable numbers of lymphocytes and neutrophils. Cases with epithelial necrosis and relatively mild inflammation may closely mimic SJS/TEN on histopathology; correlation with clinical findings, in particular the absence of a new medication and the paucity of cutaneous lesions, is necessary to exclude SJS/TEN. Recognition of the range of histopathologic features in MIRM will facilitate a more accurate and confident diagnosis of this peculiar condition, and better understanding of its pathogenic mechanism.

## Author Contributions

All authors have made substantial contributions to conception and design, or acquisition of data, or analysis and interpretation of data. All have been involved in drafting the manuscript or revising it critically for important intellectual content. All have given final approval of the version to be published, and have agreed to be accountable for all aspects of the work in ensuring that questions related to the accuracy or integrity of any part of the work are appropriately investigated and resolved.

## Ethics Statement

This study was approved by the IRB at the University of Michigan.

## Conflicts of Interest

The authors declare no conflicts of interest.

## Data Availability

The data that support the findings of this study are available on request from the corresponding author. The data are not publicly available due to privacy or ethical restrictions.
